# Diagnostic radiology training for medical students - a Brazilian multicenter survey

**DOI:** 10.31744/einstein_journal/2023AO0184

**Published:** 2023-03-07

**Authors:** Natally Horvat, Brunna Clemente de Oliveira, Daniella Braz Parente, Julia Werneck Paulino Soares de Souza, Livia Roma Barbosa, Isabel Veras Beleza, Géssica Silva Cazagrande, Rackel Silva Resende, Scott Andrew Rohren, Parth Patel, Mohamed E. Badawy, Munevver Nur Duran, Umayma Abdullatif, Serageldin Kamel, Jacob Stanietzky, Khaled M. Elsayes

**Affiliations:** 1 Memorial Sloan Kettering Cancer Center New York NY USA Memorial Sloan Kettering Cancer Center , New York , NY , USA .; 2 Alphaclin Diagnósticos Porto Velho RO Brazil Alphaclin Diagnósticos , Porto Velho , RO , Brazil .; 3 Universidade Federal do Rio de Janeiro Rio de Janeiro RJ Brazil Universidade Federal do Rio de Janeiro , Rio de Janeiro , RJ , Brazil .; 4 Universidade Estácio de Sá Rio de Janeiro RJ Brazil Universidade Estácio de Sá , Rio de Janeiro , RJ , Brazil .; 5 Universidade de Taubaté Taubaté SP Brazil Universidade de Taubaté , Taubaté , SP , Brazil .; 6 Universidade de Fortaleza Fortaleza CE Brazil Universidade de Fortaleza , Fortaleza , CE , Brazil .; 7 Universidade de Vassouras Vassouras RJ Brazil Universidade de Vassouras , Vassouras , RJ , Brazil .; 8 Universidade Federal da Fronteira Sul Chapecó SC Brazil Universidade Federal da Fronteira Sul , Chapecó , SC , Brazil .; 9 Baylor College of Medicine Houston TX USA Baylor College of Medicine , Houston , TX , USA .; 10 UTHealth Houston McGovern Medical School Houston TX USA UTHealth Houston McGovern Medical School , Houston , TX , USA .; 11 The University of Texas MD Anderson Cancer Center Houston TX USA The University of Texas , MD Anderson Cancer Center , Houston , TX , USA .; 12 University of Texas Southwestern Medical Center Dallas TX USA University of Texas Southwestern Medical Center , Dallas , TX , USA .; 13 University of Houston Houston TX USA University of Houston , Houston , TX , USA .

**Keywords:** Radiology, education, Education, medical, Students, medical, Surveys and questionnaires, Clinical competence, Health knowledge, attitudes, pratice

## Abstract

**Objective:**

This study aimed to assess diagnostic radiology training and exposure during medical school, from the perspective of medical students in Brazil.

**Methods:**

In this multicenter study approved by the Institutional Review Board, medical students from multiple universities in Brazil filled out an online questionnaire regarding their perception about diagnostic radiology training during medical school, including knowledge and use of the American College of Radiology Appropriateness Criteria and their confidence level in interpreting common radiological findings. Medical students from different regions of Brazil were sent invitations to participate in the anonymous survey through radiology group emails initiated by radiology professors and a group of ambassadors representing different institutions. Informed consent was obtained electronically at the beginning of the survey.

**Results:**

The survey demonstrated diagnostic radiology is frequently included in preclinical exams; however, radiology training during medical school was considered inadequate from the medical students´ perspective. Overall, radiological imaging teaching was provided by radiologists for more than half of the survey respondents; however, radiological imaging is frequently shown to students by non-radiologist physicians during case discussion rounds. Moreover, few respondents had a mandatory radiology training rotation during medical school.

**Conclusion:**

This Brazilian medical student survey demonstrated that from the medical students’ perspective, diagnostic radiology is an important subject in clinical practice; however, their radiology training and exposure are overall heterogeneous.

**Figure:**
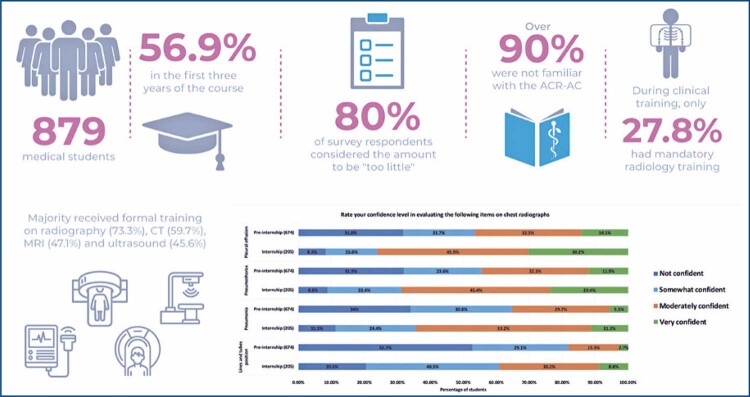

## INTRODUCTION

Brazil has the sixth largest population in the world with over 213 million inhabitants. Due to numerous public policy programs that have been put into place, the number of medical schools, mostly private, has significantly increased since the beginning of the century. In 2019, there were 335 medical schools in Brazil, and new units are opened every year. ^( 1 )^ The completion of medical school in Brazil takes 6 years, with an average 2 years of basic science instruction, 2 years of clinical instruction, and 2 years of clerkships, which are called intern years. After completion of medical school, there are several paths to practice different specialties. Unlike other systems, residency is not mandatory in Brazil, and newly graduates can enter the job market as general physicians. Most of these new doctors work in emergency medicine, intensive care, or primary care. ^( 2 )^

In recent decades, technical and scientific advances in radiology made imaging an increasingly important in clinical practice. Consequently, there is a growing need in Brazil for designing reasonable medical graduation syllabus with appropriate radiology training. Currently, formal radiology graduation training is usually incorporated into different subjects and most schools do not offer radiology as subject. Moreover, during two-year internship, radiology exposure is not required, and most schools do not offer it as an option. ^( 3 , 4 )^

In the United States, to minimize imaging overuse and encourage prudent use of imaging resources, the American College of Radiology (ACR) developed the ACR Appropriateness Criteria (ACR-AC), in which a list from the most to the least appropriate imaging exam to order is presented for an array of clinical presentations. ^( 5 )^ In line with that, the Brazilian National Curriculum Guidelines of the Medical Graduation Courses, published in 2014, established that the newly graduate must know how to order exams, based on the best scientific evidence, according to the needs of the person under their care, considering accessibility, efficiency, efficacy, and effectiveness of the studies. ^( 6 )^ Additionally, the Brazilian guidelines stated medical students should be able to identify critical imaging findings, emphasizing early diagnosis is key to improving clinical outcomes.

In the international literature, several articles have been published in the recent years, discussing radiology instruction as part of medical school training. ^( 7 - 10 )^ A comprehensive literature review of 142 articles demonstrated radiology is considered important by medical schools, albeit some do not include formal radiology training and consequently, a significant proportion of students lack knowledge of the essentials of radiology. ^( 3 )^ Moreover, while medical students considered radiology to be a valuable subject, their clinical exposure to radiology during medical school was scarce overall. ^( 10 - 12 )^

In Brazil, only a few studies have evaluated radiology syllabus, with one study demonstrating radiology training is heterogeneous across medical schools. ^( 13 )^ However, none of the Brazilian studies have evaluated the need for radiology training based on the perspective of medical students regarding their training and exposure.

## OBJECTIVE

This study aimed to assess diagnostic radiology training and exposure during medical school, from the perspective of medical students in Brazil.

## METHODS

In this multicenter study approved by the Institutional Review Boards, medical students from several universities in Brazil filled out an online questionnaire administered via the SurveyMonkey platform (SurveyMonkey Inc., San Mateo, CA, U.S.). Survey questions were designed by one board-certified radiologist with 20-year experience in medical education, and derived from prior questionnaires published in the U.S. literature. ^( 8 , 11 )^ The survey was reviewed and translated into Portuguese to adapt it to the Brazilian medical school syllabus, by three board-certified radiologists with at least 5-year experience in medical education and by two medical school students.

The questionnaire assessed the following group of characteristics: demographic data, including which fellowship program the student is planning to apply to; overview of the student’s formal diagnostic radiology training during medical school and their perspective regarding the amount of radiology education during their training; overview of the student’s diagnostic radiology exposure during medical school, including knowledge and use of the ACR Appropriateness Criteria; the student’s confidence level in interpreting common findings on chest radiographs, including position of lines and tubes, pneumonia, pneumothorax, and pleural effusion; and the student’s perception of the importance for interns to interpret findings on various imaging modalities.

The confidence level in interpreting common findings on chest radiographs was rated on a 4-point scale, as follows: 1-not confident, 2-somewhat confident, 3-moderately confident, and 4-very confident. The perception of the importance for interns to interpret findings on various imaging modalities was rated on a 4-point scale, as follows: 1- not important, 2- somewhat important, 3- moderately important, and 4- very important.

Medical students from different regions of Brazil were invited to participate in the anonymous survey through radiology group emails initiated by radiology lecturers, and a group of ambassadors representing different organizations. The requirements to complete the survey were: enrollment in a Brazilian medical school and willingness to complete the entire survey. Informed consent was obtained electronically at the beginning of the survey. Descriptive analysis was performed using the SurveyMonkey data expressed in frequency and proportions.

## RESULTS

### Demographic data

Between July 2021 and September 2021, a total of 879 Brazilian medical students from different medical schools completed the survey ( Table 1 ). Most of the respondents were female (600/879, 68.3%) and were in the first three years of medical school (500/879, 56.9%). Regarding future career plans, few considered radiology as their first option for their fellowship program application (51/879, 5.8%), the majority had not decided yet (321/879, 36.5%), 268 (30.5%) considered clinical and 239 (27.2%) surgical fellowship programs.

**Table 1:** Demographic data of medical students who responded to the survey

Demographic characteristics	n (%)
Sex	
Female	600 (68.3)
Male	276 (31.4)
Medical school year	
1 ^st^ year	127 (14.5)
2 ^nd^ year	153 (17.4)
3 ^rd^ year	220 (25.0)
4 ^th^ year	174 (19.8)
5 ^th^ year	128 (14.6)
6 ^th^ year	63 (7.2)
Other	14 (1.5)
Fellowship program to which the medical student planning to apply	
Clinical	268 (30.5)
Surgical	239 (27.2)
Radiology	51 (5.8)
Undecided	321 (36.5)

### Overview of diagnostic radiology training during medical school

Table 2 summarizes the survey results regarding diagnostic radiology training during medical school. Among the respondents who were undergoing clinical training at the time of the survey (564/879, 64.2%), 192 (34.0%) did not have dedicated diagnostic radiology training, with such training being mandatory for only 157/564 (27.8%) respondents. On the other hand, radiological images were included on preclinical exams for most of the respondents (711/879, 80.9%). Overall, radiologists ( *versus* non-radiologists or both radiologists and non-radiologists) provided almost half of medical imaging teaching (395/879, 44.9%). Among the different teaching strategies, regular lectures were the most common tool employed during radiology training (662/879, 75.3%), followed by imaging evaluation in rounds or case discussions (294/879, 33.5%), and self-guided learning with images (293/879, 33.3%). Less than one-third of respondents stated their medical school provided resources that allowed them to go through images independently (194/879, 22.1%). Very few respondents reported interacting with radiologists at least monthly during their clinical rotation (89/879, 10.1%).

**Table 2:** Overview of diagnostic radiology training during medical school and medical students’ perspective

Diagnostic radiology training	n (%)
Clinical diagnostic radiology training	
Mandatory training	157 (17.9)
Elective training	64 (7.3)
No training	192 (21.8)
Not sure	151 (17.2)
Not yet on clinical training	315 (35.8)
Radiology imaging included on preclinical exams	
Yes	711 (80.9)
No	168 (19.1)
Who provided the medical imaging teaching	
Radiologists	395 (44.9)
Non-radiologists	140 (15.9)
Radiologists and non-radiologists	148 (16.8)
None	132 (15.0)
Not sure	64 (7.3)
Radiology teaching strategies during medical school*	
Regular lectures	662 (75.3)
Imaging evaluation during rounds and case discussion	294 (33.5)
Self-guided learning with images	293 (33.3)
Problem-based small group learning dedicated to medical imaging	179 (20.4)
None	128 (14.6)
Medical school provided resources that allowed students go through the images on their own	
Yes	194 (22.1)
No	297 (33.8)
Not sure	388 (44.1)
Frequency of interaction between students and radiologists during clinical rotations	
Daily	6 (0.7)
Few times a week	21 (2.4)
Few times a month	62 (7.1)
Once or twice a year	69 (7.9)
Never	113 (12.9)
Not applicable	608 (69.2)
Students’ perception of amount of radiology education during their training	
Too little	703 (80)
Adequate	174 (19.8)
Too much	2 (0.2)

* More than one option was allowed.

### Relevance of diagnostic radiology training from the medical students’ perspective

Most respondents thought the amount of radiology education during their medical school training was “too little” (703/879, 80%). Regarding the importance for interns to interpret imaging modalities, most considered it was important for interns to independently interpret brain computed tomography (717/879, 81.7%), as well as chest radiography (829/879, 94.5%), abdominal radiography (770/879, 87.6%), and bone radiography (728/879, 82.8%) ( Table 3 ).

**Table 3:** Medical students’ perception of the importance for interns to interpret imaging modalities

Imaging modalities	Not important n (%)	Somewhat important n (%)	Moderately important n (%)	Very important n (%)
Chest radiography	2 (0.2)	10 (1.1)	36 (4.1)	829 (94.5)
Abdominal radiography	2 (0.2)	25 (2.8)	82 (9.3)	770 (87.6)
Bone radiography	1 (0.1)	14 (1.6)	135 (15.4)	728 (82.8)
Brain computed tomography	5 (0.6)	24 (2.7)	132 (15.0)	717 (81.7)

### Overview of diagnostic radiology exposure

Table 4 summarizes the survey results of the respondents’ diagnostic radiology exposure during medical school. Almost half of respondents reported that during rounds where radiological images were shown to them and discussed by non-radiologists (386/879, 43.9%). The majority of respondents had never heard of or had heard but were not familiar with the ACR Appropriateness Criteria (733/879, 83.3%), and a few of them used it at least monthly (25/879, 2.8%).

**Table 4:** Overview of medical students’ diagnostic radiology exposure during medical school

Diagnostic radiology exposure	n (%)
Scenarios where radiological images were showed to students	
On rounds while discussing with radiologists	283 (32.2)
On rounds while discussing with non-radiologists	386 (43.9)
On rounds while discussing with training physicians	178 (20.3)
During a radiology elective	46 (5.3)
Not seen	66 (7.5)
Not applicable	297 (33.8)
ACR Appropriateness Criteria knowledge	
Never heard of	446 (50.7)
Heard of it but not familiar	287 (32.6)
Somewhat familiar	138 (15.7)
Very familiar	8 (0.9)
Frequency of ACR Appropriateness Criteria use on clinical rotations	
Not applicable	361 (41.1)
Never used	465 (52.9)
Few times a year	28 (3.2)
Few times a month	11 (1.3)
Few times a week	11 (1.3)
Daily	3 (0.3)
Imaging modalities with formal training*	
Conventional radiography	644 (73.3)
Fluoroscopy	128 (14.6)
Ultrasound	401 (45.6)
Computed tomography	525 (59.7)
Magnetic resonance imaging	414 (47.1)
Nuclear medicine (including positron emission tomography)	136 (15.5)
None of them	215 (24.5)
Radiological topics with formal training*	
Radiation safety	143 (16.3)
Imaging algorithms	60 (6.8)
Normal radiographic anatomy	559 (63.6)
Abnormal radiographic anatomy	544 (61.9)
Common findings on radiography ( *e.g* ., central lines, pacemakers)	338 (38.5)
None of them	45 (5.1)
Diseases with formal imaging training*	
Bone fractures	484 (55.1)
Pneumonia	596 (67.8)
Pleural effusion	589 (67.0)
Brain hemorrhage	379 (43.1)
Pneumothorax	583 (66.3)
None of the above	183 (20.8)

* More than one option was allowed.

ACR: American College of Radiology.

With regards to the imaging modalities for which the respondents received formal training, survey results showed the respondents had predominantly conventional radiography (644/879, 73.3%) and computed tomography (525/879, 59.7%) training, followed by magnetic resonance imaging (414/879, 47.1%) and ultrasound (401/879, 45.6%) training. Few of the respondents had fluoroscopy (128/879, 14.6%) or nuclear medicine (136/879, 15.5%) training. Almost one-third of respondents did not have any formal training on imaging modalities (215/879, 24.5%).

Regarding formal training in radiology-related topics, the survey revealed normal and abnormal radiographic anatomy was taught to 559/879 (63.6%) and 544/879 (61.9%) respondents, respectively. Common findings on radiography, such as central lines and pacemakers, were taught to 338/879 (38.5%) respondents, while radiation safety and imaging algorithms were taught to 143/879 (16.3%) and 60/879 (6.8%) respondents. With regard to diseases, more than half of participants had formal training in bone fractures (484/879, 55.1%), pneumonia (596/879, 67.8%), pleural effusion (589/879, 67.0%), and pneumothorax (583/879, 66.3%); while 379/879 (43.1%) had training in brain hemorrhage.

### Imaging interpretation confidence on chest conventional radiography

**Figure 1:**
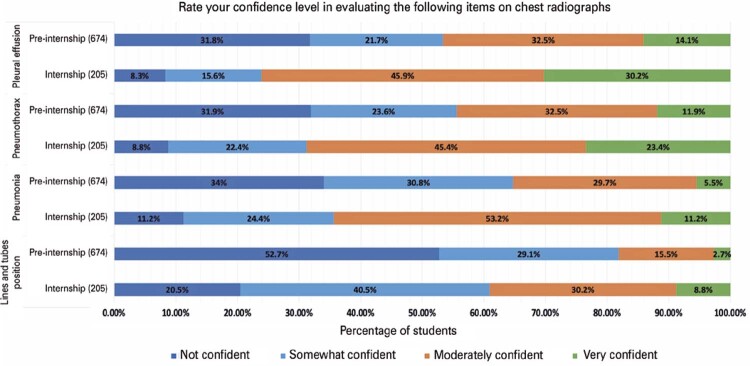
Graphic showing the rate of chest radiograph interpretation among medical students on pre-internship and during the internship

Almost half of respondents were not confident in interpreting the position of lines and tubes on chest X-rays (397, 45.2%), while the majority were at least somewhat confident in evaluating pneumonia (626, 71.2%), pneumothorax (645, 73.5%), and pleural effusion (648, 73.7%) as shown in table 5 . Overall, the confidence level increased among students during internship ( Figure 1 ).

**Table 5:** Confidence level of medical students in interpreting chest conventional radiography

Chest conventional radiography findings	Not confident n (%)	Somewhat confident n (%)	Moderately confident n (%)	Very confident n (%)
Position of lines and tubes	397 (45.2)	279 (31.8)	166 (18.9)	36 (4.1)
Pneumonia	252 (28.7)	257 (29.3)	309 (35.2)	60 (6.8)
Pneumothorax	233 (26.5)	205 (23.4)	312 (35.5)	128 (14.6)
Pleural effusion	231 (26.3)	178 (20.3)	313 (35.6)	157 (17.9)

## DISCUSSION

Our survey demonstrated diagnostic radiology is frequently included in preclinical exams; however, radiology training during medical school was considered inadequate from the perspective of medical students. A total of 80% of survey respondents considered the amount of radiology education to be “too little”, similarly to prior studies from other countries. ^( 9 , 12 , 14 )^ Overall, radiological imaging teaching was provided by one board-certified radiologist for more than half of the survey respondents; however, radiological imaging is frequently shown to students by non-radiologists during case discussion rounds. Moreover, only 20% of respondents had a mandatory radiology training rotation during medical school, similar to what has been published in literature for medical students in Egypt. ^( 14 )^

Previous studies demonstrated that over 20% of medical students denied having any clinical training in radiology, only 23% declared that a radiology internship is required, and 15% chose radiology as an elective rotation. ^( 14 )^ Our results are in line with a study carried out in Scotland, which showed most medical schools do not have adequate radiology training, ^( 9 )^ confirming not only the underrepresentation of this subject in the medical syllabus, but also the lack of standardization on radiology teaching.

Additionally, less than 20% of medical students had formal radiation safety training and this is a concern for patients, physicians, and staff in several departments of the hospital. Knowledge on radiation safety and protection allow physicians to reduce the risk of unnecessary radiation exposure for both patients and medical providers.

Most of the respondents of our survey (more than 90%) were not familiar with the ACR-AC and did not use it on clinical rotations. This figure is higher than that found in a similar study conducted by Badawy et al., in Egyptian medical schools. ^( 14 )^ But it must be taken into account that, unlike Badawy et al., most participants in this study were in the preclinical years of medical school.

More than two thirds of respondents of our survey reported having training on conventional radiography and on common chest conditions, such as pneumothorax, pleural effusion, and pneumonia. Additionally, many of them were at least somewhat confident in interpreting the most common chest conditions on x-rays. Although most respondents considered it is very important for interns to interpret brain computed tomography, less than a half reported formal training in brain hemorrhage. Overall, the confidence level in interpretating radiographs increased among students on internship. Finally, only 10.1% of respondents had at least monthly interaction with radiologists.

Noticeably, there is a gap between what students expected and what they are taught. Our results may help inform the tailoring of educational initiatives to overcome this gap in medical school teaching. Several strategies may be implemented to improve medical imaging teaching, such as e-learning, flipped classrooms, problem-solving scenarios, and integrated medical training during all years of the medical school training. ^( 9 , 15 , 16 )^ Regardless of the type of strategic plan, virtual platforms are extremely beneficial and recommended, particularly after COVID-19 pandemic, considering its wide use and acceptance. ^( 17 , 18 )^ In line with that, previous studies evaluated effectiveness of online teaching among medical students and showed virtual platforms are feasible and well accepted. ^( 19 , 20 )^ A virtual platform approach can also reach many medical students, allows for multidisciplinary discussion, and increases opportunities for interaction between medical students and radiologists.

Our study also has some limitations. Although 80% of survey respondents considered the amount of radiology education “too little”, most of the participants in this study were in the preclinical years of medical school.

Radiology has been recognized as a relevant skill; however, few studies have shown objective improvements in medical student outcomes related to radiology training. Chew et al. showed that small group radiology teaching significantly improved anatomy scores in their end of year examination. ^( 21 )^ Further studies are warranted to assess and quantify the clinical impact of different radiology educational strategies. Ultimately, this will guide universities, national committees, and boards throughout the world to develop and implement improvements in medical school education.

## CONCLUSION

This Brazilian medical student survey demonstrated diagnostic radiology is an important discipline in clinical practice from perspective of medical students; however, their radiology training and exposure are overall heterogeneous. Further studies are needed to explore different educational strategies and their impact on medical students’ clinical knowledge of key radiological concepts.
